# Peripheral Artery Disease as a Predictor of Coronary Artery Disease in Patients Undergoing Coronary Angiography

**DOI:** 10.7759/cureus.15094

**Published:** 2021-05-18

**Authors:** Ashok Kumar, Shehar Bano, Umama Bhurgri, Jatender Kumar, Ahmed Ali, Suman Dembra, Love Kumar, Simra Shahid, Dua Khalid, Amber Rizwan

**Affiliations:** 1 Medicine, Liaquat University of Medical and Health Sciences, Jamshoro, PAK; 2 Internal Medicine, University of Health Sciences, Lahore, PAK; 3 Internal Medicine, Liaquat University of Medical and Health Sciences, Jamshoro, PAK; 4 Internal Medicine, Jinnah Sindh Medical University, Karachi, PAK; 5 Infectious Diseases Department, University of Louisville, Louisville, USA; 6 Medicine, Peoples University of Medical and Health Sciences for Women, Nawabshah, PAK; 7 Medicine, Jinnah Sindh Medical University, Karachi, PAK; 8 Family Medicine, Jinnah Post Graduate Medical Center, Karachi, PAK

**Keywords:** peripheral artery disease, coronary artery disease, predictive tool, angiography, cardiology

## Abstract

Background

Peripheral artery disease (PAD) may be a useful tool to predict coronary artery disease (CAD) in patients undergoing coronary angiography. If proven that PAD can be a good predictor of CAD, it can help in early and cost-effective diagnosis of CAD.

Methodology

This observational study was conducted from January 2020 to February 2021 in the cardiology unit of a tertiary care hospital. Participants older than 40 years, with a history of uncontrolled hypertension and unstable angina, who warranted the need of angiography were enrolled in study. After enrollment and recording history, these cases were assessed for the presence of PAD based on ankle brachial index (ABI). ABI values less than 0.9 were labelled as participants with PAD. Then these cases underwent coronary angiography at the same institute, and the presence of greater than 50% stenosis of any coronary vessel on angiography was taken as positive CAD.

Results

In this study, PAD was identified in 152 (62.8%) participants. A total of 165 (68.1%) participants had greater than 50% stenosis on angiography. Out of 152 participants with ABI less than 0.9, 140 had greater than 50% stenosis on angiography. In total, 90 participants had ABI more than 0.9, of which 35 participants had greater than 50% stenosis. Sensitivity of PAD in predicting coronary artery stenosis was 80.0% (95% confidence interval [CI]: 73.30%-85.66%), specificity was 82.09% (95% CI: 70.80%-90.39%), and accuracy was 80.58% (95% CI: 75.02%-85.37%).

Conclusions

Our study demonstrated that the sensitivity, specificity, and accuracy of PAD in predicting coronary artery stenosis were significant. Hence, we conclude that PAD can be an excellent predictor of CAD by helping in early and cost-effective diagnosis of CAD.

## Introduction

Peripheral artery disease (PAD) is an occlusive disease affecting the arterial supply of the lower limb [1,2]. It affects approximately 120 million people worldwide and accounts for almost 26% of the global burden of cardiovascular diseases [3]. The prevalence of the disease increases with increasing age and affects almost one-fourth of people over the age of 55 years [4]. The underlying pathogenesis of PAD is consistent with that of coronary artery disease (CAD), where atherosclerotic changes in the arteries lead to the disease manifestation [5,6]. The REACH dataset found that almost one-third of the patients with CAD also had PAD while almost two-thirds of PAD patients had a coexisting CAD or cerebrovascular disease [7,8]. Similarly, the GenePAD study also reported that almost one-fourth of the patients with CAD had a concomitant PAD, supporting common pathophysiology and risk [9]. This correlation could stem from their common risk factors which include diabetes mellitus (DM), hypertension, hyperlipidemia, and lifestyle-related factors such as smoking and sedentary and stressful life [10]. PAD may also be a useful predictor for CAD in patients undergoing coronary angiography. A study conducted in Brazil indicated 95.4% specificity of ankle brachial index (ABI) for predicting significant CAD in patients with an ABI of ≤0.87. It also showed positive predictive value of 75.9%, while negative predictive value of 71.6% [11]. Currently, there is no local study available investigating the correlation between PAD and coronary stenosis. If proven that PAD can be a good predictor of CAD, it can help in the early and cost-effective diagnosis of CAD.

## Materials and methods

This observational study was conducted from January 2020 to February 2021 in the cardiology unit of a tertiary care hospital. Participants older than 40 years, with a history of uncontrolled hypertension and unstable angina, who warranted the need of angiography were enrolled in study. A total of 242 participants were enrolled in the study via consecutive convenient non-probability sampling from the outpatient department. The detailed demographic data such as age, gender, and presence of comorbidities were noted in a self-structured questionnaire. After enrollment and recording history, these cases were assessed for PAD based on ABI. ABI values less than 0.9 were labelled as participants with PAD. Then these cases underwent coronary angiography at the same institute. The presence of greater than 50% stenosis of any coronary vessel on angiography was taken as positive CAD.

Statistical Package for Social Sciences® software version 23.0 (IBM Corp., Armonk, NY, USA) was used for data analysis. For numerical variables, data such as age were expressed as mean ± standard deviation. Frequencies and percentages were used for categorical variables. An online calculator (MedCalc, Ostend, Belgium) was used to calculate the sensitivity, specificity, positive predictive value, and negative predictive value for PAD in predicting coronary stenosis.

## Results

The mean age of participants in the study was 50 ± 11 years. Hyperlipidemia was the most common comorbidity (66.9%), followed by type two DM (46.3%). Overall, 13.2% participants mentioned a past history of myocardial infarction (Table 1).

**Table 1:** Characteristics of participants enrolled in the study. ACEi: angiotensin-converting enzyme inhibitors; ARB: Angiotensin receptor blocker; CCB: calcium channel blocker

Characteristics	Frequency (Percentages)
Age in years (mean ± standard deviation)	50 ± 11
Gender	
Male	141 (58.3%)
Female	101 (41.7%)
History of comorbidities	
Type 2 diabetes mellitus	112 (46.3%)
Hyperlipidemia	162 (66.9%)
Smoker	89 (36.8%)
Hyperuricemia	84 (34.7%)
Past and family history	
Family history of myocardial infarction	41 (16.9%)
Previous history of myocardial infarction	32 (13.2%)
Current medication	
Statins	201 (83.1%)
ACEi	92 (38.0%)
ARBs	151 (62.4%)
Diuretics	88 (36.4%)
CCBs	41 (16.9%)
Anti-platelet drug (aspirin, clopidogrel, or both)	198 (81.8%)

In this study, PAD was identified in 152 (62.8%) participants. In total, 175 (72.3%) participants had greater than 50% stenosis in angiography. Sensitivity of PAD in predicting coronary artery stenosis was 80.0% (95% confidence interval [CI]: 73.30%-85.66%), specificity was 82.09% (95% CI: 70.80%-90.39%), and accuracy was 80.58% (95% CI: 75.02%-85.37%) (Table 2).

**Table 2:** Prediction of coronary artery disease using ankle brachial index. CI: confidence interval

Ankle brachial index		Greater than 50% stenosis		Sensitivity % (CI)	Specificity % (CI)	Positive predictive value	Negative predictive value	Accuracy
		Yes	No					
Less than 0.9	152	140	12	80.00 (73.30-85.66)	82.09 (70.80-90.39)	92.11 (87.42-95.14)	61.11 (53.38-68.32)	80.58 (75.02-85.37)
More than 0.9	90	35	55					

## Discussion

Systemic atherosclerosis leads to grave manifestations such as PAD and CAD. The development of PAD and CAD clinically overlap due to their shared risk factors. In the present study, hyperlipidemia and type II DM were among the significant comorbidities in these patients. Hyperglycemia and dyslipidemia play a significant role in the pathogenesis of atherosclerosis. Old age, hypertension, DM, hyperlipidemia, and smoking are among the main predictors of PAD in patients with CAD [12,13].

In our study, 62.8% of patients were found to have PAD in association with CAD. ABI has been shown to be a non-invasive, sensitive, specific, and cost-effective tool for lower limb vascular assessment and evaluating atherosclerosis in lower limbs. It represents the ratio of the ankle to brachial systolic pressure and is calculated by dividing the higher systolic pressure of the dorsalis pedis and tibialis posterior vessels at the ankle with that measured in the brachial artery in both arms [14]. An ABI below 0.9 is considered a powerful independent marker of coronary morbidity and mortality [15]. According to Sabedotti et al., ABI of <0.87 had a specificity of 95.4% and a positive predictive value of 75.9% [16].

Diagnosing CAD requires substantial healthcare resources and the expertise of a cardiologist. In developing countries and remote regions, such expertise and healthcare resources may not be available readily. The ABI is widely used for screening purposes for CAD in different clinical settings by healthcare professionals, ranging from general medical practitioners to cardiologists and specialist vascular technicians [17]. Performing and interpreting the results of ABI does not require the expertise of a specialist. Furthermore, its low cost and easy accessibility make ABI a useful tool in the diagnosis and risk stratification of CAD suspects in low- and middle-income countries [16].

Numerous studies have provided evidence that a significant proportion of patients with CAD had coexistent PAD. Saleh et al. proposed that PAD was prevalent in 14.7% of patients with CAD, which was significantly higher than that in patients with normal coronaries (p-value:  < 0.001) [12]. Hur et al. also suggested a positive correlation of severe PAD with CAD in 72% of patients [18]. Another study showed that one out of six patients with CAD had a coexistent unrecognized PAD despite being under the care of a cardiovascular specialist [19]. Another study also found a strong correlation, with 46.88% occurrence of CAD in PAD-positive cases [20].

The high prevalence of PAD with CAD and the high positive predictive value of ABI suggest that a patient with PAD could be screened for CAD with high suspicion and pretest probability. However, the diagnosis of CAD is highly dependent on tests including electrocardiogram, stress electrocardiogram, myocardial perfusion imaging, coronary angiography, and other cardiac imaging modalities. ABI can add to the pretest probability of CAD, but it cannot substitute any of the above testing modalities.

Our study suggests that routine determination of ABI and correct diagnosis and supervision of patients with PAD can help in the early and cost-effective diagnosis of CAD and prevent any coronary stenosis-related event in the future. Therefore, the diagnosis of PAD in patients should prompt the clinicians to have a high clinical index of suspicion for possible CAD, especially if they have hypertension, dyslipidemia, diabetes, and other risk factors.

This study has its limitations. Because it was a single-center study, the sample was less diverse and limited. Keeping in mind the aforementioned facts, further large-scale studies are required to establish the correlation between PAD and CAD.

## Conclusions

Our study demonstrated that the sensitivity, specificity, and accuracy of PAD in predicting coronary artery stenosis was significant. Hence, we conclude that with a high positive predictive value, PAD can be an excellent predictor of CAD. It can help in the early and cost-effective diagnosis of CAD. However, further large-scale, multi-centered studies are needed to study their correlation.
